# Supplementary material for: Coupling of ribosome biogenesis and translation initiation in human mitochondria
Source: Nat Commun. 2025 Apr 17;16:3641. doi: 10.1038/s41467-025-58827-x (PMC12003892; doi:10.1038/s41467-025-58827-x)
Supplement: Supplementary file 1 — Supplementary Information [file 41467_2025_58827_MOESM1_ESM.pdf]

Supplementary information

SUPPLEMENTARY FIGURES

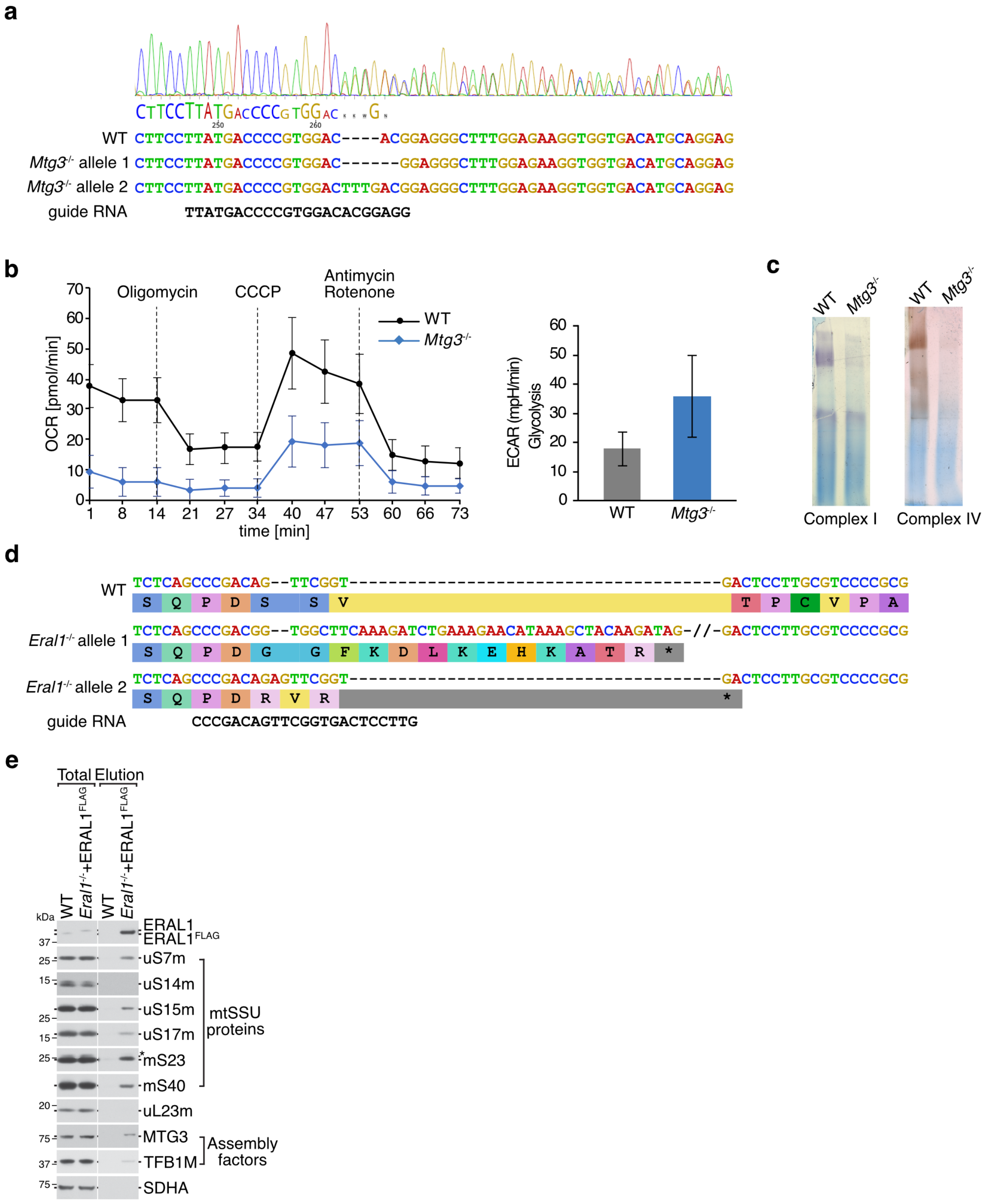

Supplementary Fig. 1

(a) Genomic sequencing of the *Mtg3*$^{-/-}$ cell line. Genomic DNA was isolated from wild type and *Mtg3*$^{-/-}$ cl.1 and the guide RNA target region amplified via PCR. PCR products were analyzed via TOPO cloning and subsequent sequencing. Chromatogram shows the target region with the corresponding sequence of the wild type cells and the two mutated alleles in the *Mtg3*$^{-/-}$. **(b)** Real time respiratory measurements. Left: Representative graph of the oxygen consumption rate (OCR) analyzed for wild

type and *Mtg3*$^{-/-}$ cells. Reagents allowing the measurement of different metabolic conditions were added at indicated time points. Right: quantification of glycolysis activity. (mean ± SD, n= 16 technical replicates). **(c)** In gel activity analysis of complex I and complex IV. Solubilized mitochondria from wild type and *Mtg3*$^{-/-}$ cells were loaded on a 3 %-10 % polyacrylamide gel and BN-PAGE was performed. Subsequently, gels were incubated in complex I or IV activity solutions, respectively. Similar results were obtained in n ≥ 3 biologically independent experiments. **(d)** Genomic sequencing of the *Eral1*$^{-/-}$ cell line used for transfection with an ERAL1^{FLAG} construct for subsequent FLAG immunoprecipitation. Sequencing result was obtained as described in (a). The KO cell line was generated by using a guide RNA targeting the first exon of the *Eral1* gene. A two bp insertion and a 123 bp insertion in the two alleles lead to premature stop codons and truncated proteins. **(e)** Western blot analysis of mitoribosome complexes co-purified via ERAL1^{FLAG}. FLAG-immunoprecipitation was performed with lysed mitochondria from wild type cells and *Eral*1$^{-/-}$ cells inducibly expressing ERAL1^{FLAG}. Samples (total = 3 %, eluate = 100 %) were subsequently analyzed via western blotting with antibodies as indicated. Similar results were obtained in n ≥ 3 biologically independent experiments.

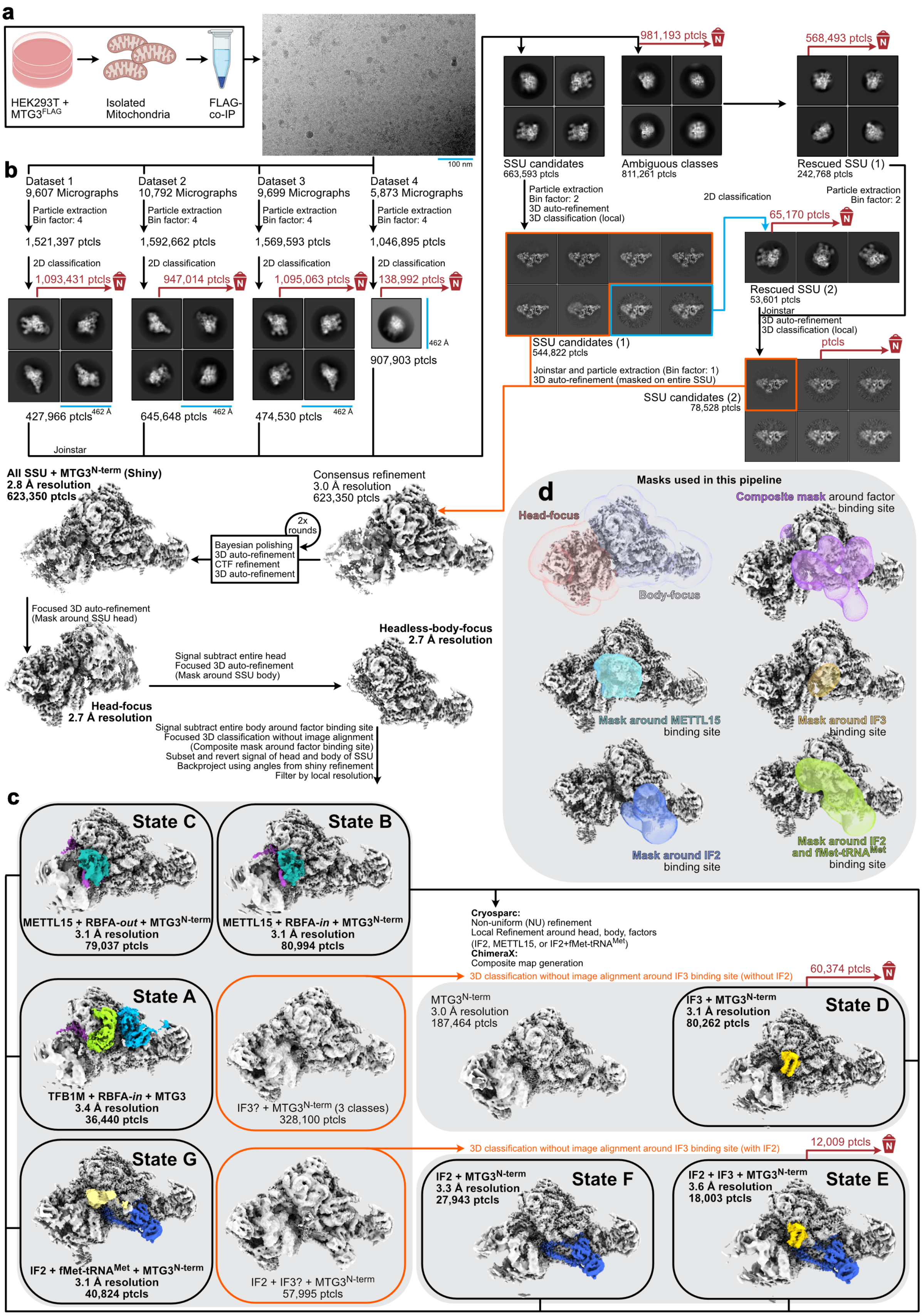

Supplementary Fig. 2 Cryo-EM reconstruction pipeline. **(a)** Isolation of mitoribosomes and exemplary cryo-EM micrograph. **(b)** Cryo-EM processing workflow for structure determination. Exemplary aligned class averages from 2D and initial 3D classifications are shown. **(c)** Local resolution filtered maps of the final subsets. Additional densities for the factors are highlighted in the following

colors: MTG3: light blue, TFB1M: lime green, mtRBFA: indigo, METTL15: turquoise, mtIF3: yellow, mtIF2: blue, fMet-tRNA^{Met}: light yellow. **(d)** Local masks as utilized in the processing workflow. Created in BioRender. Finke, F. (2025) https://BioRender.com/l09i305.

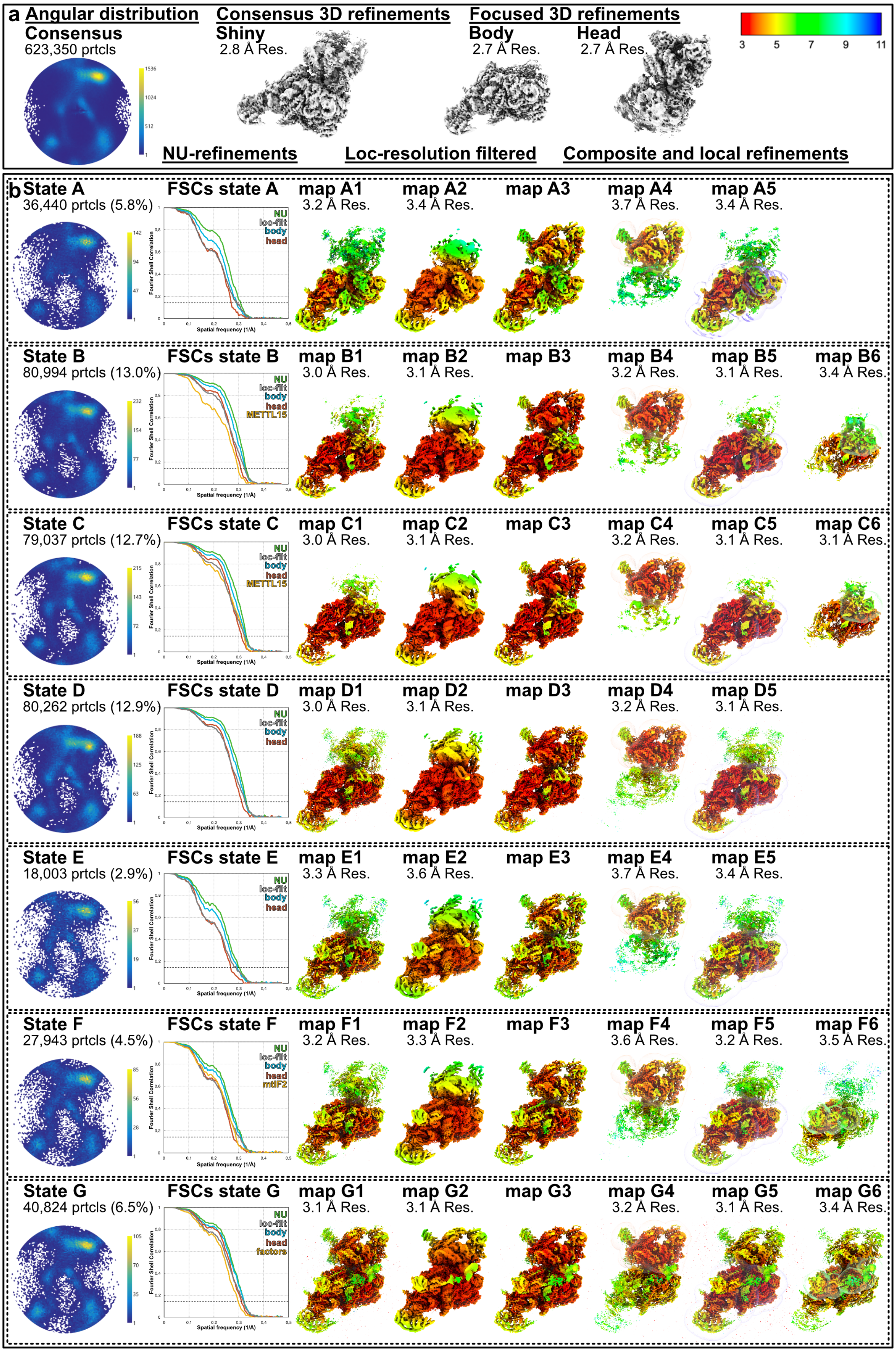

Supplementary Fig. 3 Overall-refined maps for each state.

(a) Consensus refinement (unfocused, body-focused, head-focused) and angular distribution plot for all mtSSU particles. **(b)** Local resolution estimation of non-uniform refinements from cryoSPARC, local resolution filtered maps (B-Factor 0) from Relion of each state, as well as composite maps together with corresponding local refinements from cryoSPARC together with corresponding FSC-plots and angular distribution plots for each state.

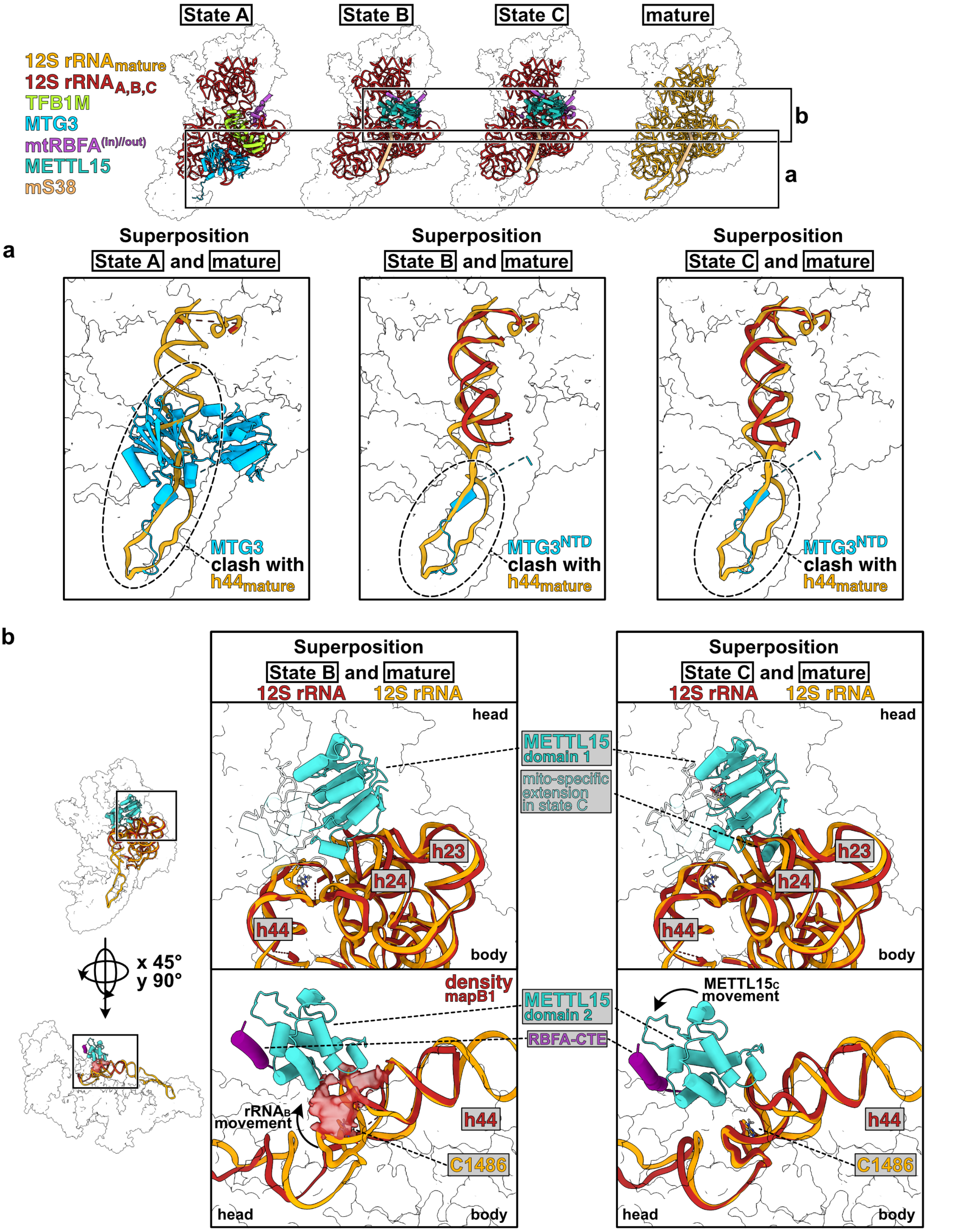

Supplementary Fig. 4 Structural comparison of all three assembly states with mature mtSSU. **(a)** Close-up view of the foot region of states A-C superimposed with mature mtSSU (PDB: 7PO2; [19] was taken for better visual comparison since h44 is fully resolved). MTG3 from state A and remaining MTG^{NTD} from states B and C are shown, and clashes with mature h44 are indicated. **(b)** Zoomed-in views of state B and C superimposed with mature mtSSU (PDB: 7PO2; [19]). Depiction of protein-rRNA-interactions between the CTD of METTL15 (domain 1) and h23 and h24 and depiction of protein-rRNA-interactions between the NTD of METTL15 (domain 2) and h44. Residues around C1486 are not modelled in state B but rRNA density from map B3 is shown as red transparent surface. Residue

C1486 from mature mtSSU is shown. (**a-b**) Depiction as followed: states A-C vs PDB: 7PO2; [19]: surface: not shown vs white, 12S rRNA: red vs orange; MTG3: blue, METTL15: turquoise, mtRBFA-CTE: indigo.

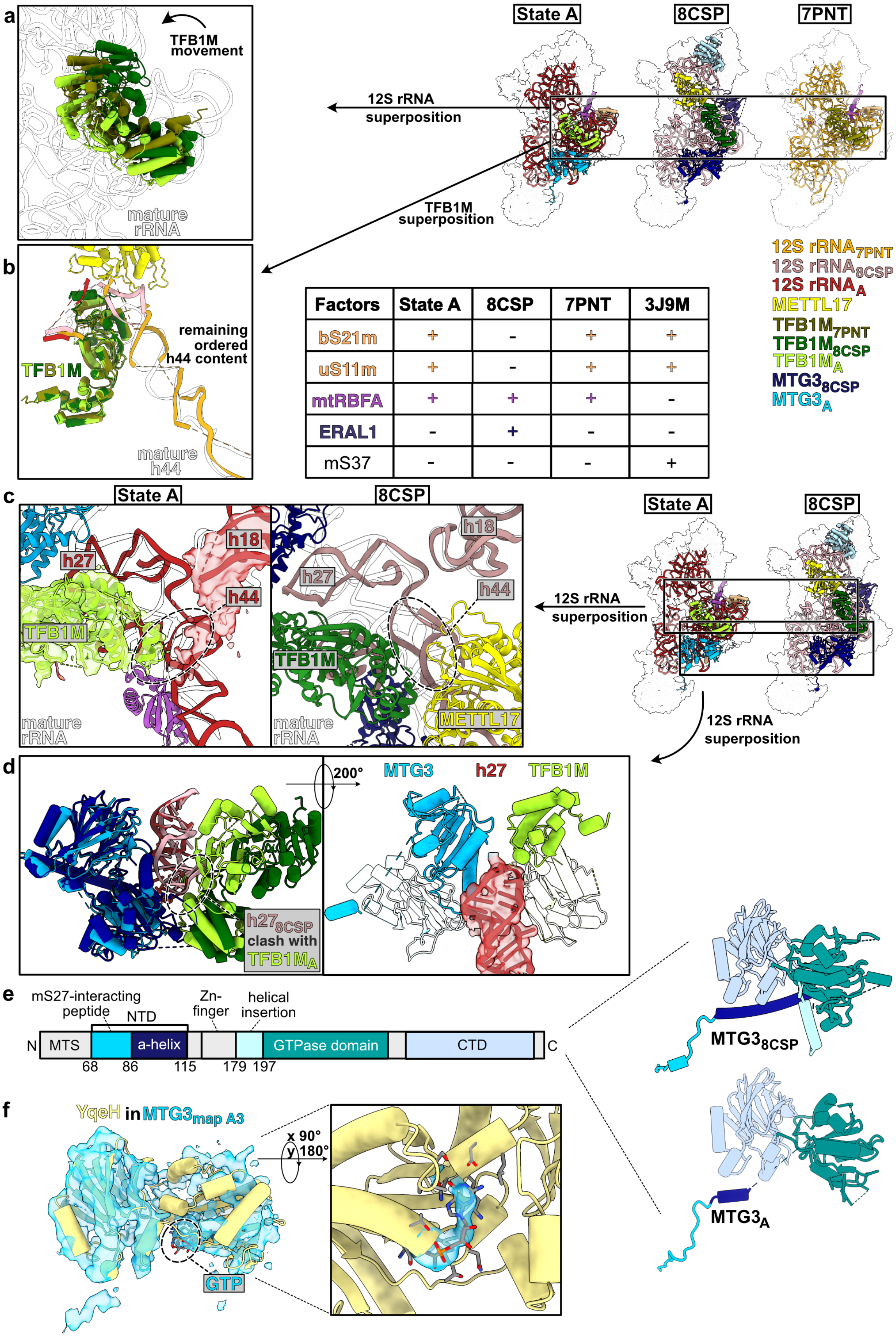

Factors	State A	8CSP	7PNT	3J9M
bS21m	+	-	+	+
uS11m	+	-	+	+
mtRBFA	+	+	+	-
ERAL1	-	+	-	-
mS37	-	-	-	+

Supplementary Fig. 5 Structural details of state A.
(a) Composition of the shoulder region in state A and resulting re-orientation of TFB1M compared to previously reported states (PDB: 7PNT; [19], PDB: 8CSP; [20]). **(b)** Comparison of resolved h44-h45 linker regions in the published states in (**a**) compared to state A in this study by superposition of TFB1M from all states. **(c)** Composition of h44-h45 linker region in state A (density from map A3) and the previously published state (PDB: 8CSP; [20]) **(d)** Close-up view of h27 in its mature conformation from our state A in density from map A3 (right) vs superposition with immature h27 from already known state (left) (PDB: 8CSP; [20]). **(e)** Schematic depiction of the primary structure of MTG3 and highlighted areas as depicted in published (PDB: 8CSP; [20]) and our models. **(f)** Superposition of YqeH (PDB: 3EC1; [25]) in MTG3 density from map A3 (state A) and close-up view of YqeH GTP-binding site containing GDP overlayed with density from map A3.

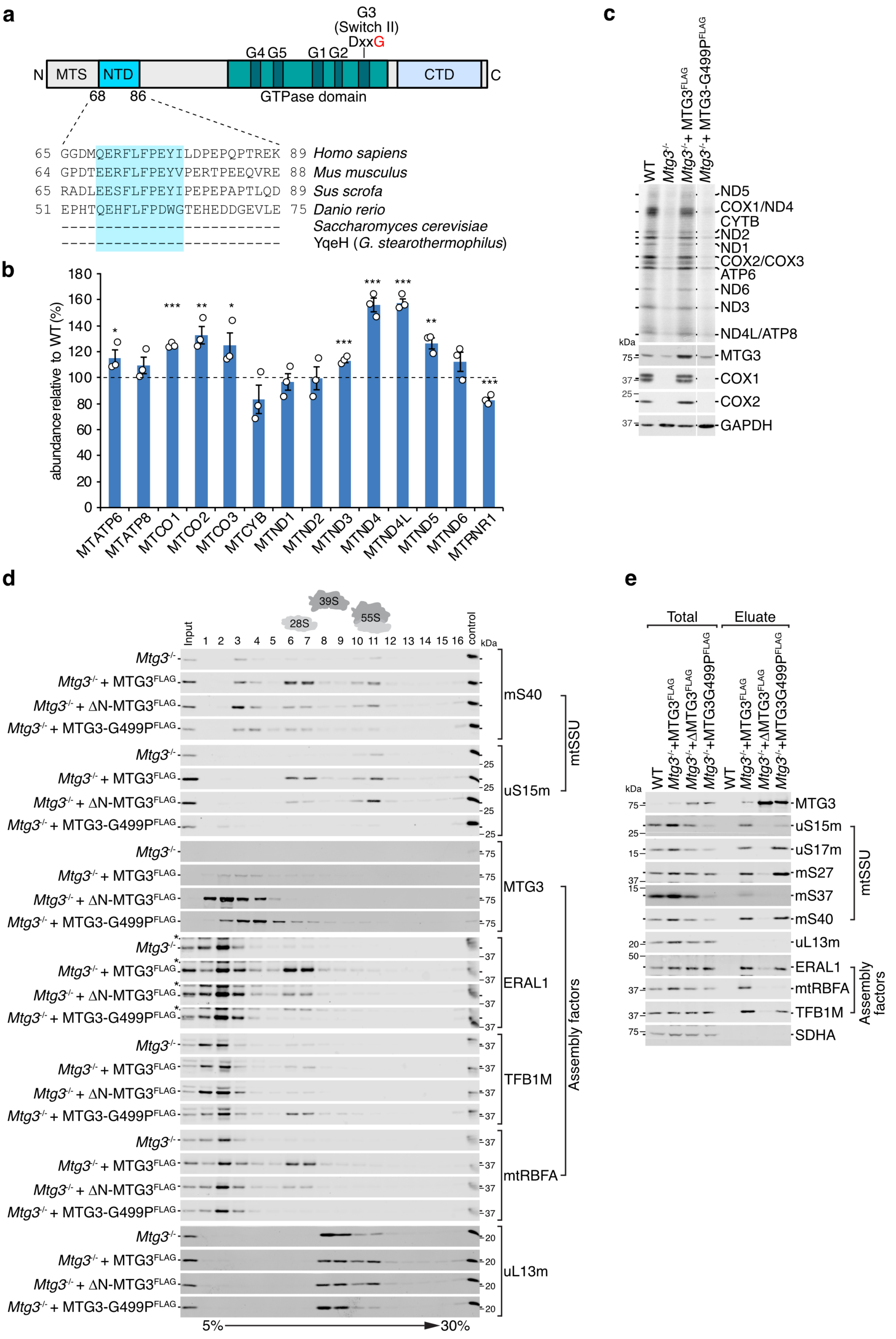

Supplementary Fig. 6 Mutational analysis of NTD and GTPase domain of MTG3

(a) Schematic representation of MTG3 domains and sequence alignment of the N-terminal region. MTS (mitochondrial targeting sequence), N-terminal domain, zink finger domain and GTPase domain are indicated as boxes. The location of G499P mutation in the G3 motif is indicated in red. Sequence alignment of the conserved N-terminal region with the 10 aa (transparent blue box) interacting with mS27 and displacing h44 is shown below. **(b)** Abundance of total mt-mRNAs is similar in the *Mtg3*$^{-/-}$ + ΔN-MTG3^{FLAG} cell line in comparison to wild type MTG3^{FLAG}. Total mitochondrial lysates ('input' from sucrose gradients from (Fig. 5c)) were used to isolate RNA and perform NanoString analysis. Level of mt-mRNAs were normalized to 16S-rRNA and RNA abundance in the *Mtg3*$^{-/-}$ + ΔN-MTG3^{FLAG} cell line was calculated relative to *Mtg3*$^{-/-}$ + MTG3^{FLAG} cell line (n=3 biologically independent experiments shown as mean ± SEM; individual data points are shown as circles). Statistical analysis was performed as two-sample one-tailed Student's t-test. Significance was defined as $*p \leq 0.05$, $**p \leq 0.01$, $***p \leq 0.001$. **(c)** MTG3-G499P^{FLAG} mutant cannot restore mitochondrial translation. Translation of mtDNA-encoded proteins in wild type, *Mtg3*$^{-/-}$ and *Mtg3*$^{-/-}$ cell lines expressing full length (MTG3^{FLAG}) or mutated MTG3 (MTG3-G499P^{FLAG}), respectively, were analyzed using [^{35}S]Methionine *de novo* incorporation and visualized via autoradiography and western blotting. GAPDH was used as a loading control. Similar results were obtained in n ≥ 3 biologically independent experiments. **(d)** Comparison of monosome and mtSSU formation in MTG3 mutants. Mitoplasts (500 µg) were isolated from *Mtg3*$^{-/-}$ and *Mtg3*$^{-/-}$ cell lines expressing full length (MTG3^{FLAG}), ΔN-MTG3^{FLAG} or MTG3-G499P^{FLAG}, respectively. Mitoribosomal complexes were separated via sucrose density gradient centrifugation and collected fractions (1-16) were analyzed via western blotting with indicated antibodies against MRPs and assembly factors. Similar results were obtained in n ≥ 3 biologically independent experiments. **(e)** MTG3-G499P^{FLAG} can co-iolated early-stage mtSSU intermediates. FLAG-immunoprecipitation was performed with wild type cells and *Mtg3*$^{-/-}$ cells expressing MTG3FLAG, ΔN-MTG3^{FLAG} or MTG3-G499P, respectively. Samples were analyzed using western blotting and antibodies as indicated (total = 3 %, eluate = 100 %). Similar results were obtained in n ≥ 3 biologically independent experiments.

SUPPLEMENTARY TABLES

Supplementary Table 1: Cryo-EM data collection for all states, and refinement and validation for state A-C.

**Table 1. Cryo-EM data collection, refinement, and validation statistics**						
Data collection and processing						
Magnification	81,000x		Pixel size (Å)		1.05	
Voltage (kV)	300		Symmetry imposed		C1	
Electron exposure ($e^-/Å^2$)	40		Initial particle images (no.)		5,730,547	
Defocus range (μm)	0.6-1.6					
	Map A1	Map A2	Map A3	Map A4	Map A5	
EMDB ID	51066	51053	51083	51064	51065	
Final particle images (no.)	36,440	36,440	–	36,440	36,440	
Map resolution (Å)	3.2	3.4	–	3.7	3.4	
FSC threshold	0.143	0.143	–	0.143	0.143	
Map sharpening *B* factor ($Å^2$)	–	–	–	–	–	
	Map B1	Map B2	Map B3	Map B4	Map B5	Map B6
EMDB ID	51063	51052	51084	51061	51062	51060
Final particle images (no.)	80,994	80,994	–	80,994	80,994	80,994
Map resolution (Å)	3.0	3.1	–	3.2	3.1	3.4
FSC threshold	0.143	0.143	–	0.143	0.143	0.143
Map sharpening *B* factor ($Å^2$)	–	–	–	–	–	–
	Map C1	Map C2	Map C3	Map C4	Map C5	Map C6
EMDB ID	51059	51051	51085	51057	51058	51056
Final particle images (no.)	79,037	79,037	–	79,037	79,037	79,037
Map resolution (Å)	3.0	3.1	–	3.2	3.1	3.1
FSC threshold	0.143	0.143	–	0.143	0.143	0.143
Map sharpening *B* factor ($Å^2$)	–	–	–	–	–	–
Refinement	PDB: 9G5B		PDB: 9G5C		PDB: 9G5D	
Model resolution (Å) (A-C2)	3.0		2.8		2.8	
FSC threshold	0.143		0.143		0.143	
Model composition						
Non-hydrogen atoms	68,043		67,559		68,810	
Protein residues	6,226		5,965		6,049	
Nucleotide residues	817		891		918	
Ligands	ATP: 1, ZN: 1, FES: 2, MG: 12		ATP: 1, ZN: 1, FES: 2, K: 4, MG: 23		ATP: 1, ZN: 1, FES: 2, NAD: 1, K: 1, MG: 10	
Mean *B* factors ($Å^2$) (A-C2)						
Protein	95.82		71.32		73.64	
Nucleotide	121.73		78.01		79.32	
Ligand	112.03		83.43		87.23	
RMSDs						
Bond lengths (Å)	0.004		0.003		0.004	
Bond angles (°)	0.810		0.722		0.727	
Validation						
MolProbity score	1.46		1.28		1.27	
Clashscore	5.20		5.30		5.06	
Poor rotamers (%)	0.00		0.00		0.02	
Ramachandran plot						
Favored (%)	96.89		98.22		98.14	
Allowed (%)	3.11		1.78		1.84	
Disallowed (%)	0.02		0.00		0.02	

Supplementary Table 2: Cryo-EM data refinement and validation for state D-G.

Table 2. Cryo-EM data refinement, and validation statistics						
Data processing						
	Map D1	Map D2	Map D3	Map D4	Map D5	
EMDB ID	51047	51046	52117	52063	52062	
Final particle images (no.)	80,262	80,262	–	80,262	80,262	
Map resolution (Å)	3.0	3.1	–	3.2	3.1	
FSC threshold	0.143	0.143	–	0.143	0.143	
Map sharpening *B* factor (Å^2)	–60.8	–	–	–60.4	–56.0	
	Map E1	Map E2	Map E3	Map E4	Map E5	
EMDB ID	51045	51043	52118	52065	52064	
Final particle images (no.)	18,003	18,003	–	18,003	18,003	
Map resolution (Å)	3.3	3.6	–	3.7	3.4	
FSC threshold	0.143	0.143	–	0.143	0.143	
Map sharpening *B* factor (Å^2)	–36.8	–	–	–41.1	–35.8	
	Map F1	Map F2	Map F3	Map F4	Map F5	Map F6
EMDB ID	51055	51050	51086	51049	51054	51048
Final particle images (no.)	27,943	27,943	–	27,943	27,943	27,943
Map resolution (Å)	3.2	3.3	–	3.6	3.2	3.5
FSC threshold	0.143	0.143	–	0.143	0.143	0.143
Map sharpening *B* factor (Å^2)	–43.6	–	–	–47.3	–42.1	–65.1
	Map G1	Map G2	Map G3	Map G4	Map G5	Map G6
EMDB ID	51042	51039	52119	52067	52066	52068
Final particle images (no.)	40,824	40,824	–	40,824	40,824	40,824
Map resolution (Å)	3.1	3.1	–	3.2	3.1	3.4
FSC threshold	0.143	0.143	–	0.143	0.143	0.143
Map sharpening *B* factor (Å^2)	–52.5	–	–	–52.3	–49.2	–59.6

Refinement	PDB: 9HFM	PDB: 9HFN	PDB: 9G5E	PDB: 9HFO
Model resolution (Å) (D-G2)	2.8	2.9	2.9	2.8
FSC threshold	0.143	0.143	0.143	0.143
Model composition				
Non-hydrogen atoms	66,106	69,310	68,649	71,379
Protein residues	5,756	6,169	6,088	6,217
Nucleotide residues	897	897	897	979
Ligands	ATP: 1, ZN: 1, FES: 2, K: 1, MG: 44	ATP: 1, GTP: 1 ZN: 1, FES: 2, K: 1, MG: 45	ATP: 1, GTP: 1 ZN: 1, FES: 2, K: 1, MG: 45	ATP: 1, GTP: 1 ZN: 1, FES: 2, K: 1, MG: 45
Mean *B* factors (Å^2) (D-G2)				
Protein	146.73	103.71	102.61	127.45
Nucleotide	97.43	94.80	94.02	77.39
Ligand	69.12	44.69	44.24	126.82
RMSDs				
Bond lengths (Å)	0.005	0.004	0.003	0.004
Bond angles (°)	0.808	0.811	0.798	0.746
Validation				
MolProbity score	1.34	1.47	1.35	1.26
Clashscore	4.16	5.63	4.39	4.96
Poor rotamers (%)	0.00	0.00	0.00	0.00
Ramachandran plot				
Favored (%)	97.27	97.07	97.30	98.58
Allowed (%)	2.70	2.93	2.68	1.40
Disallowed (%)	0.04	0.00	0.02	0.02

Supplementary Table 3: Events in the biogenesis pathway for each resolved mtSSU state in this study.

Matured helices and presence of certain assembly and translation initiation factors are marked with a plus symbol. States for comparison are taken from [19] (PDB: 7PO2) and [53] (PDB: 6GAW).

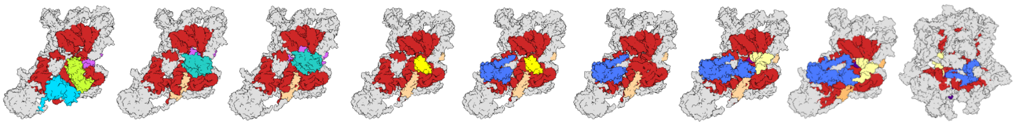

	State A	State B	State C	State D	State E	State F	State G	7PO2	6GAW
**matured?**									
h18	-	+	+	+	+	+	+	+	+
h24	-	-	-	+	+	+	+	+	+
h45	-	-	-	+	+	+	+	+	+
h44-h45	-	-	-	+	+	+	+	+	+
h44	-	-	-	-	-	-	-	+	+
**present?**									
MTG3	+	-	-	-	-	-	-	-	-
MTG3$^{N\text{-term}}$	+	+	+	+	+	+	+	-	-
mS38	-	+	+	+	+	+	+	+	+
RBFA$^{(in)/(out)}$	+	+	+	-	-	-	-	-	-
METTL15	-	+	+	-	-	-	-	-	-
mS37	-	-	-	+	+	+	+	+	+
mtIF3	-	-	-	+	+	-	-	-	-
mtIF2	-	-	-	-	+	+	+	+	+
fMet-tRNA^{Met}	-	-	-	-	-	-	+	+	+
mRNA^{AUG}	-	-	-	-	-	-	+	+	+
mtLSU	-	-	-	-	-	-	-	-	+

Supplementary Table 4: Key Resource Table

Reagent or resource	Supplier	Identifier
**Antibodies**		
Rabbit polyclonal anti-C4orf14 (MTG3, 1:1000)	Invitrogen	PA5-57420
Rabbit polyclonal anti-ERAL1 (1:2000)	ProteinTech	11478-1-AP
Rabbit polyclonal anti-TFB1M (1:1000)	ProteinTech	16604-1-AP
Rabbit polyclonal anti-RBFA (1:1000)	Novus Biologicals	NBP1-88471
Rabbit polyclonal anti-NSUN4 (1:1000)	ProteinTech	16320-1-AP
Rabbit polyclonal anti-mtIF2 (1:1000)	This study	PRAB5207
Rabbit polyclonal anti-mtIF3 (1:1000)	ProteinTech	14219-1-AP
Mouse monoclonal anti-Calnexin (1:500 000)	ProteinTech	66903-1-Ig; 2A2C6
Mouse monoclonal anti-SDHA (1:20 000)	Invitrogen	459200; 2E3GC12FB2AE2
Mouse monoclonal anti-GAPDH (1:100 000)	Santa Cruz	sc-32233
Rabbit polyclonal anti-COX1 (1:2000)	[39]	PRAB5121
Mouse monoclonal anti-COX2 (1:1000)	Abcam	Ab110258; 12C4F12
Rabbit polyclonal anti-ATP6 (1:5000)	[42]	PRAB5159
Rabbit polyclonal anti-bS1m (1:1000)	ProteinTech	16378-1-AP
Rabbit polyclonal anti-uS5m (1:5000)	Proteintech	16428-1-AP
Rabbit polyclonal anti-uS7m (1:1000)	Sigma-Aldrich	HPA 023007
Rabbit polyclonal anti-uS10m (1:1000)	ProteinTech	16030-1-AP
Rabbit polyclonal anti-uS14m (1:1000)	ProteinTech	16301-1-AP
Rabbit polyclonal anti-uS15m (1:5000)	ProteinTech	17106-1-AP
Rabbit polyclonal anti-uS17m (1:1000)	ProteinTech	18881-1-AP
Rabbit polyclonal anti-mS22 (1:5000)	ProteinTech	10984-1-AP
Rabbit polyclonal anti-mS23 (1:10000)	ProteinTech	18345-1-AP
Rabbit polyclonal anti-mS25 (1:5000)	ProteinTech	15277-1-AP
Rabbit polyclonal anti-mS27 (1:10000)	ProteinTech	17280-1-AP
Rabbit polyclonal anti-mS29 (1:1000)	ProteinTech	10276-1-AP
Rabbit polyclonal anti-mS34 (1:5000)	ProteinTech	15166-1-AP
Rabbit polyclonal anti-mS35 (1:5000)	ProteinTech	16457-1-AP
Rabbit polyclonal anti-mS37 (1:1000)	ProteinTech	11728-1-AP
Rabbit polyclonal anti-mS40 (1:5000)	ProteinTech	16139-1-AP
Rabbit polyclonal anti-uL13m (1:5000)	Proteintech	16241-1-AP
Rabbit polyclonal anti-uL23m (1:10 000)	[39]	PRAB1716
Rabbit polyclonal anti-POLRMT (1:1000)	ProteinTech	17748-1-AP
Rabbit polyclonal anti-LRPPRC (1:2000)	ProteinTech	21175-1-AP
Goat IgG anti-rabbit IgG (H+L)-HRPO (1:5000)	dianova	111-035-144
Goat IgG anti-mouse IgG (H+L)-HRPO (1:5000)	dianova	115-035-146
IRDye 800CW Donkey anti-mouse (1:10 000)	LI-COR	926-32212
IRDye 800CW Donkey anit-rabbit (1:10 000)	LI-COR	926-32213
**Oligonucleotides**		
Primer: Generation of the FLAG-tagged version of MTG3 Forward: 5'-CTTTCTGATATCCCACCATGCTGCCCGCTCGCCTACCGTTC-3'	This study; Microsynth	
Primer: Generation of the FLAG-tagged version of MTG3	This study; Microsynth	

Reverse: 5'-CTCTCCCTCGAGCTACTTATCGTCGTCATCCTTGTAATCTACATTTATCTTTCCTTTCTTCTTCC-3'		
Primer: Generation of the FLAG-tagged version of mtIF3 Forward: 5'-CTTTCTGATATCCCACCATGGCTGCTCTTTTTCTAAAGAGGTT-3'	This study; Microsynth	
Primer: Generation of the FLAG-tagged version of mtIF3 Reverse: 5'-CTCTCCCTCGAGCTACTTATCGTCGTCATCCTTGTAATCCTGATGCAGAACATTTGATTC-3'	This study; Microsynth	
Primer: Generation of the FLAG-tagged mutant version of MTG3 (ΔN-MTG3) Forward: 5'-TTGGAGAAGGTGGTGACATGCTGGATCCGGAG-3'	This study; Microsynth	
Primer: Generation of the FLAG-tagged mutant version of MTG3 (ΔN-MTG3) Reverse: 5'-GGTTGCGGCTCCGGATCCAGCATGTCACCA-3'	This study; Microsynth	
Primer: Generation of the FLAG-tagged mutant variant of MTG3 (G499P) Forward: 5'-CAC TGG TTT TAT GAC ACC CCT **CC**A ATT ACA AAA-3'	This study; Microsynth	
Primer: Generation of the FLAG-tagged mutant variant of MTG3 (G499P) Reverse: 5'-ACA ATT TTC TTT TGT AAT T**GG** AGG GGT GTC ATA-3'	This study; Microsynth	
Primer: Generation of the FLAG-tagged variant of ERAL1 Forward: 5`-CTTTCTGATATCCCACCATGGCTGCCCCCAGCTGGCG-3`	This study; Microsynth	
Primer: Generation of the FLAG-tagged variant of ERAL1 Reverse: 5'-CTCTCCCTCGAGCTACTTATCGTCGTCATCCTTGTAATCCTTGAGGAGCTTCACAGAGAG-3'	This study; Microsynth	
Guide RNA: targeting Exon 1 of MTG3 5'-TTATGACCCCGTGGACACGG-3'	This study; IDT	
Guide RNA: targeting Exon 1 of ERAL1 5'-CCCGACAGTTCGGTGACTCCTTG-3'	This study; IDT	
Probe for northern blot: targeting MTRNR1 (12S rRNA) 5'-TCGATTACAGAACAGGCTCCTCTAG-3'	[27]	
Probe for northern blot: targeting MTRNR2 (16S rRNA) 5'- GTTTGGCTAAGGTTGTCTGGTAGTA-3'		
Probe for northern blot: targeting MTCO1 5'- GTCAGTTGCCAAAGCCTCCGATTATG-3'		
Probe for northern blot: targeting 18S-rRNA 5'- TTTACTTCCTCTAGATAGTCAAGTTCGACC-3'		
**NanoString sequences**		
CTTCATCAGGGTTTGCTGAAGATGGCGGTATATAGGCTGAGCAAGAGGTGCCTCAAGACCTAAGCGACAGCGTGACCTTGTTTCA	IDT, [39, 42]	Mito_JCM__008.1:591_T001
CTTTCTTAATTGGTGGCTGCTTTTAGGCCTACTATGGGTGTTAAATTTTTCATCCTCTTCTTTTCTTGGTGTTGAGAAGATGCTC		Mito_JCM__009.1:481_T002

GTTCTTGTGTTGTGATAAGGGTGGAGAGGTTAAAGGAG CCACTTATTAGTCACAATTCTGCGGGTTAGCAGGAAGG TTAGGGAAC		HSMT_ND1.1: 461_T003
CCTGCTATGATGGATAAGATTGAGAGAGTGAGGAGAA GGCTTACGTTTAGCTGTTGAGATTATTGAGCTTCATCAT GACCAGAAG		HSMT_ND2.1: 444_T004
GCGGAGGTGAAATATGCTCGTGTGTCTACGTCTATTCC TACTGTAAATATCAAAGACGCCTATCTTCCAGTTTGAT CGGGAAACT		HSMT_COX1.1 :873_T005
TCGTCTGTTATGTAAAGGATGCGTAGGGATGGGAGGG CGATGAGGACTAGCGAACCTAACTCCTCGCTACATTCC TATTGTTTTC		HSMT_COX2.1 :216_T006
TATTTTTATGGGCTTTGGTGAGGGAGGTAGGTGGTAGT TTGTGTTTAATACCAATTTGGTTTTACTCCCCTCGATTA TGCGGAGT		HSMT_ATP8.1: 82_T007
GTGGGCTAGGGCATTTTTAATCTTAGAGCGAAAGCCTA TAATCACTGTGCCTTTCGGGTTATATCTATCATTTACTT GACACCCT		HSMT_ATP6.1: 331_T008
ATGTTGAGCCGTAGATGCCGTCGGAAATGGTGAAGGG AGACTCGAAGTACCAACAGCCACTTTTTTCCAAATTT TGCAAGAGCC		HSMT_COX3.1 :539_T009
AAGGTAATAGCTACTAAGAAGAATTTTATGGAGAAAG GGACGCGGGCGGGCACCGTGTGGACGGCAACTCAGAG ATAACGCATAT		HSMT_ND3.1: 135_T010
TATTCCTTCTAGGCATAGTAGGGAGGATATGAGGTGTG AGCGATATACTACCTGGAGTTTATGTATTGCCAACGAG TTTGTCTTT		HSMT_ND4L.1 :58_T011
ACTGTGAGTGCGTTCGTAGTTTGAGTTTGCTAGGCAGA ATAGTAATGAGGCAGATAAGGTTGTTATTGTGGAGGAT GTTACTACA		HSMT_ND4.1: 967_T012
TGCGGTTTCGATGATGTGGTCTTTGGAGTAGAAACCTG TGAGGAAAGGTACTTCCTTCCTGTGTTCCAGCTACAAA CTTAGAAAC		HSMT_ND5.1: 1147_T013
CCTCAGGATACTCCTCAATAGCCATCGCTGTAGTATAT CCAAAGACAACCCATAAAATTGGTTTTGCCTTTCAGCA ATTCAACTT		HSMT_ND6.1: 191_T014
GAGGTCTGCGGCTAGGAGTCAATAAAGTGATTGGCTTA GTGGGCGAAATACTGGTCAAGACTTGCATGAGGACCC GCAAATTCCT		HSMT_CYTB.1 :946_T015
AGGGCAGGGACTTAATCAACGCAAGCTTATGACCCGC ACTTACTGGGAATCTTTCGTTGGGACGCTTGAAGCGCA AGTAGAAAAC		NR_003286.2:1 640_T016
TGCTTAGCTTCCGAGATCAGACGAGATCGGGCGCGTTC AGGGCCAGCAGACCTGCAATATCAAAGTTATAAGCGC GT		NR_023379.1:8 _T017
CGAAAGCCATGACCTCCGATCACTCTACACCTTGACCT AACGTCTTTACGTGGGTACTTGCGCTTACTTTGTAGC		Mito_JCM__00 8.1:591_ProbeB
CGAAAGCCATGACCTCCGATCACTCTTCAGTTATATGT TTGGGATTTTTAGGTAGTGGGTGTTGAGCTTGAACG		Mito_JCM__00 9.1:481_ProbeB
CGAAAGCCATGACCTCCGATCACTCGATAAATCATATT ATGGCCAAGGGTCATGATGGCAGGAGTAATCAGAGGT		HSMT_ND1.1: 461_ProbeB
CGAAAGCCATGACCTCCGATCACTCAGTATGCTAAGAT TTTGCGTAGCTGGGTTTGGTTTAATCCACCTCAACTG		HSMT_ND2.1: 444_ProbeB

CGAAAGCCATGACCTCCGATCACTCCGAGTCAGCTAAA TACTTTGACGCCGGTGGGGATAGCGATGATTATGGTA		HSMT_COX1.1:873_ProbeB
CGAAAGCCATGACCTCCGATCACTCAGTACCATTGGTG GCCAATTGATTTGATGGTAAGGGAGGGATCGTTGACC		HSMT_COX2.1:216_ProbeB
CGAAAGCCATGACCTCCGATCACTCAGCGAACAGATTT TCGTTCATTTTGGTTCTCAGGGTTTGTTATAATTTTT		HSMT_ATP8.1:82_ProbeB
CGAAAGCCATGACCTCCGATCACTCATAATAACTAGTA TGGGGATAAGGGGTGTAGGTGTGCCTTGTGGTAAGAA		HSMT_ATP6.1:331_ProbeB
CGAAAGCCATGACCTCCGATCACTCAGTTGAGCCAATA ATGACGTGAAGTCCGTGGAAGCCTGTGGCTACAAAAA		HSMT_COX3.1:539_ProbeB
CGAAAGCCATGACCTCCGATCACTCGGGCTCATGGTAG GGGTAAAAGGAGGGCAATTTCTAGATCAAATAATAAG		HSMT_ND3.1:135_ProbeB
CGAAAGCCATGACCTCCGATCACTCGAGTGGGTGTTGA GGGTTATGAGAGTAGCTATAATGAACAGCGATAGTAT		HSMT_ND4L.1:58_ProbeB
CGAAAGCCATGACCTCCGATCACTCGCTATTAGTGGGA GTAGAGTTTGAAGTCCTTGAGAGAGGATTATGATGCG		HSMT_ND4.1:967_ProbeB
CGAAAGCCATGACCTCCGATCACTCGTAGCGATGAGA GTAATAGATAGGGCTCAGGCGTTTGTGTATGATATGTT		HSMT_ND5.1:1147_ProbeB
CGAAAGCCATGACCTCCGATCACTCCGCTAACCCCACT AAAACACTCACCAAGACCTCAACCCCTGACCCCCATG		HSMT_ND6.1:191_ProbeB
CGAAAGCCATGACCTCCGATCACTCATGGTAAAAGGG TAGCTTACTGGTTGTCCTCCGATTCAGGTTAGAATGAG		HSMT_CYTB.1:946_ProbeB
CGAAAGCCATGACCTCCGATCACTCGCCTCACTAAACC ATCCAATCGGTAGTAGCGACGGGCGGTGTGTACAA		NR_003286.2:1640_ProbeB
CGAAAGCCATGACCTCCGATCACTCCGGTCTCCCATCC AAGTACTAACCAGGCCCGACCC		NR_023379.1:8_ProbeB
**Experimental models: cell lines**		
HEK293-Flp-In T-Rex	ThermoFisher Scientific	R78007
HEK293-Flp-In T-Rex-*Mtg3*$^{-/-}$	This study	
HEK293-Flp-In T-Rex-MTG3^{FLAG}	This study	
HEK293-Flp-In T-Rex-*Mtg3*$^{-/-}$ +MTG3^{FLAG}	This study	
HEK293-Flp-In T-Rex-*Mtg3*$^{-/-}$ + ΔN-MTG3^{FLAG}	This study	
HEK293-Flp-In T-Rex-*Mtg3*$^{-/-}$ + MTG3-G499P^{FLAG}	This study	
HEK293-Flp-In T-Rex-mtIF3^{FLAG}	This study	
HEK293-Flp-In T-Rex-*Eral1*$^{-/-}$ +ERAL1^{FLAG}	This study	
**Recombinant DNA**		
pOG44 Flp-Recombinase Expression Vector	ThermoFisher Scientific	V600520
pcDNA5/FRT/TO	ThermoFisher Scientific	V6520-20
**Chemicals**		
T4 Polynucleotide Kinase (T4 PNK)	ThermoFisher Scientific	EK0031
QuikChange Lightning Site-Directed Mutagenesis Kit	Agilent Technologies	210519-5
TOPO TA Cloning® Kit	ThermoFisher Scientific	45-0030
OneShot® TOP10 Chemically Competent Cells	ThermoFisher Scientific	C404004
L-[^{35}S]methionine	Hartmann Analytic	SCM-01

Adenosine 5'-triphosphate, [γ-^{32}P]	Hartmann-Analytic	SRP-501
Emetine dihydrochloride hydrate	Sigma-Aldrich	219282
Lipofectamine 3000	Invitrogen	L3000-015
Alt-R® CRISPR-Cas9 tracrRNA, ATTO™ 550	Integrated DNA technologies	1075927
Alt-R® S.p. Cas9 Nuclease V3	Integrated DNA technologies	1081058
TRIzol® Reagent	Ambion	15596018
Anti-FLAG M2 Affinity Gel	Sigma-Aldrich	A2220
FLAG-peptide	Sigma-Aldrich	F3290-4MG
Glutaraldehyde	Electron microscopy sciences	16220
**Deposited data**		
mtSSU state A, map A1	This study	EMD-51066
mtSSU state A, map A2	This study	EMD-51053
mtSSU state A, map A3	This study	EMD-51083
mtSSU state A, map A4	This study	EMD-51064
mtSSU state A, map A5	This study	EMD-51065
mtSSU state B, map B1	This study	EMD-51063
mtSSU state B, map B2	This study	EMD-51052
mtSSU state A, map B3	This study	EMD-51084
mtSSU state A, map B4	This study	EMD-51061
mtSSU state A, map B5	This study	EMD-51062
mtSSU state A, map B6	This study	EMD-51060
mtSSU state C, map C1	This study	EMD-51059
mtSSU state C, map C2	This study	EMD-51051
mtSSU state C, map C3	This study	EMD-51085
mtSSU state C, map C4	This study	EMD-51057
mtSSU state C, map C5	This study	EMD-51058
mtSSU state C, map C6	This study	EMD-51056
mtSSU state D, map D1	This study	EMD-51047
mtSSU state D, map D2	This study	EMD-51046
mtSSU state E, map E1	This study	EMD-51045
mtSSU state E, map E2	This study	EMD-51043
mtSSU state F, map F1	This study	EMD-51055
mtSSU state F, map F2	This study	EMD-51050
mtSSU state F, map F3	This study	EMD-51086
mtSSU state F, map F4	This study	EMD-51049
mtSSU state F, map F5	This study	EMD-51054
mtSSU state F, map F6	This study	EMD-51048
mtSSU state G, map G1	This study	EMD-51042
mtSSU state G, map G2	This study	EMD-51039
mtSSU state A model	This study	PDB: 9G5B
mtSSU state B model	This study	PDB: 9G5C
mtSSU state C model	This study	PDB: 9G5D
mtSSU state F model	This study	PDB: 9G5E
comparable MTG3-TFB1M-bound-mtSSU model	[20]	PDB: 8CSP
comparable TFB1M-mtRBFA(in)-bound-mtSSU model	[19]	PDB: 7PNT
comparable METTL15-mtRBFA(out)-bound-mtSSU model	[19]	PDB: 7PNX-Z
comparable mtIF3-bound-mtSSU model; starting model	[19]	PDB: 7PO1
comparable mtIF2-mtIF3-bound-mtSSU model	[26]	PDB: 6RW5

comparable IC-mtSSU model	[19]	PDB: 7PO2
Comparable mature mitoribosome model	[2]	PDB: 3J9M
**Software and Algorithm**		
Fiji ImageJ	[43]	
nSolver software	NanoString	v.4.0.70
SerialEM	[44]	v.4.0
Warp	[45]	v1.0.9
RELION	[46]	v3.1.0
cryoSPARC	[47]	v4.4.1
UCSF ChimeraX	[48, 49]	v1.5
COOT	[50]	v0.9.8.1
Phenix real space refine	[51]	v1.20.1
ISOLDE	[52]	
