# Supplementary material for: Coupling of ribosome biogenesis and translation initiation in human mitochondria
Source: Nat Commun. 2025 Apr 17;16:3641. doi: 10.1038/s41467-025-58827-x (PMC12003892; doi:10.1038/s41467-025-58827-x)
Supplement: Supplementary file 2 — Reporting Summary [file 41467_2025_58827_MOESM2_ESM.pdf]

nature portfolio

Corresponding author(s): Ricarda Richter-Dennerlein, Hauke Hillen

Last updated by author(s): Mar 19, 2025

# Reporting Summary

Nature Portfolio wishes to improve the reproducibility of the work that we publish. This form provides structure for consistency and transparency in reporting. For further information on Nature Portfolio policies, see our Editorial Policies and the Editorial Policy Checklist.

## Statistics

For all statistical analyses, confirm that the following items are present in the figure legend, table legend, main text, or Methods section.

| n/a | Confirmed | |
|---|---|---|
| ☐ | ☒ | The exact sample size (*n*) for each experimental group/condition, given as a discrete number and unit of measurement |
| ☐ | ☒ | A statement on whether measurements were taken from distinct samples or whether the same sample was measured repeatedly |
| ☐ | ☒ | The statistical test(s) used AND whether they are one- or two-sided<br>*Only common tests should be described solely by name; describe more complex techniques in the Methods section.* |
| ☒ | ☐ | A description of all covariates tested |
| ☒ | ☐ | A description of any assumptions or corrections, such as tests of normality and adjustment for multiple comparisons |
| ☐ | ☒ | A full description of the statistical parameters including central tendency (e.g. means) or other basic estimates (e.g. regression coefficient) AND variation (e.g. standard deviation) or associated estimates of uncertainty (e.g. confidence intervals) |
| ☐ | ☒ | For null hypothesis testing, the test statistic (e.g. *F*, *t*, *r*) with confidence intervals, effect sizes, degrees of freedom and *P* value noted<br>*Give P values as exact values whenever suitable.* |
| ☒ | ☐ | For Bayesian analysis, information on the choice of priors and Markov chain Monte Carlo settings |
| ☒ | ☐ | For hierarchical and complex designs, identification of the appropriate level for tests and full reporting of outcomes |
| ☒ | ☐ | Estimates of effect sizes (e.g. Cohen's *d*, Pearson's *r*), indicating how they were calculated |

*Our web collection on statistics for biologists contains articles on many of the points above.*

## Software and code

Policy information about availability of computer code

| | |
|---|---|
| Data collection | Serial EM v4.0 |
| Data analysis | Warp v1.0.9, CryoSPARC v4.4.1, RELION v3.1.0, WinCOOT v0.9.8.1, PHENIX v1.20.1, ChimeraX 1.5.1 with Isolde, nSolver software v.4.0.70, Fiji ImageJ |

For manuscripts utilizing custom algorithms or software that are central to the research but not yet described in published literature, software must be made available to editors and reviewers. We strongly encourage code deposition in a community repository (e.g. GitHub). See the Nature Portfolio guidelines for submitting code & software for further information.

## Data

Policy information about availability of data

All manuscripts must include a data availability statement. This statement should provide the following information, where applicable:

- Accession codes, unique identifiers, or web links for publicly available datasets
- A description of any restrictions on data availability
- For clinical datasets or third party data, please ensure that the statement adheres to our policy

The electron density reconstructions and structure coordinates were deposited with the Electron Microscopy Database (EMDB), and with the Protein Data Bank (PDB) under accession codes 9G5B [http://doi.org/10.2210/pdb9G5B/pdb] and EMD-51083 [https://www.ebi.ac.uk/pdbe/entry/emdb/EMD-51083] (State A), 9G5C [http://doi.org/10.2210/pdb9G5C/pdb] and EMD-51084 [https://www.ebi.ac.uk/pdbe/entry/emdb/EMD-51084] (State B), 9G5D [http://doi.org/10.2210/pdb9G5D/pdb] and EMD-51085 [https://www.ebi.ac.uk/pdbe/entry/emdb/EMD-51085] (State C), 9HFM [http://doi.org/10.2210/pdb9HFM/pdb] and EMD-52117 [https://

www.ebi.ac.uk/pdbe/entry/emdb/EMD-52117] (State D), 9HFN [http://doi.org/10.2210/pdb9HFN/pdb] and EMD-52118 [https://www.ebi.ac.uk/pdbe/entry/emdb/EMD-52118] (State E), 9G5E [http://doi.org/10.2210/pdb9G5E/pdb] and EMD-51086 [https://www.ebi.ac.uk/pdbe/entry/emdb/EMD-51086] (State F), 9HFO [http://doi.org/10.2210/pdb9HFO/pdb] and EMD-52119 [https://www.ebi.ac.uk/pdbe/entry/emdb/EMD-52119] (State G). The following atomic coordinates were used in this study: 8CSP [http://doi.org/10.2210/pdb8CSP/pdb] (human mtSSU assembly intermediate with MCAT, METTL17, TFB1M, MTG3), 8CSR [http://doi.org/10.2210/pdb8CSR/pdb] (human mtSSU assembly intermediate with MCAT, METTL17, TFB1M), 7PNX [http://doi.org/10.2210/pdb7PNX/pdb] (human mtSSU assembly intermediate with mtRBFA, METTL15), 7PO1 [http://doi.org/10.2210/pdb7PO1/pdb] (human mtSSU with mtIF3), 7PO2 [http://doi.org/10.2210/pdb7PO2/pdb] (human mtSSU with mtIF2, fMet-tRNAMet, mRNA), 3J9M [http://doi.org/10.2210/pdb3J9M/pdb] (human mature mitoribosome), 6GAW [http://doi.org/10.2210/pdb6GAW/pdb] (human mitoribosome with mtIF2, fMet-tRNAMet, mRNA), 6RW5 [https://doi.org/10.2210/pdb6RW5/pdb] (human mitochondrial 28S ribosome in complex with mitochondrial IF2 and IF3). Source data are provided with this manuscript.

## Research involving human participants, their data, or biological material

Policy information about studies with human participants or human data. See also policy information about sex, gender (identity/presentation), and sexual orientation and race, ethnicity and racism.

| | |
|---|---|
| Reporting on sex and gender | n.a. |
| Reporting on race, ethnicity, or other socially relevant groupings | n.a. |
| Population characteristics | n.a. |
| Recruitment | n.a. |
| Ethics oversight | n.a. |

Note that full information on the approval of the study protocol must also be provided in the manuscript.

# Field-specific reporting

Please select the one below that is the best fit for your research. If you are not sure, read the appropriate sections before making your selection.

[x] Life sciences [ ] Behavioural & social sciences [ ] Ecological, evolutionary & environmental sciences

For a reference copy of the document with all sections, see nature.com/documents/nr-reporting-summary-flat.pdf

# Life sciences study design

All studies must disclose on these points even when the disclosure is negative.

| | |
|---|---|
| Sample size | No statistical methods were used to predetermine sample size. All biochemical experiments were replicated three or more times. |
| Data exclusions | No data were excluded from the analyses. |
| Replication | All attempts at replication were successful. |
| Randomization | Samples were not allocated to groups. |
| Blinding | Investigators were not blinded during data acquisition and analysis because it is not a common procedure for the methods employed. |

# Reporting for specific materials, systems and methods

We require information from authors about some types of materials, experimental systems and methods used in many studies. Here, indicate whether each material, system or method listed is relevant to your study. If you are not sure if a list item applies to your research, read the appropriate section before selecting a response.

## Materials & experimental systems

| n/a | Involved in the study | |
|---|---|---|
| [ ] | [x] | Antibodies |
| [ ] | [x] | Eukaryotic cell lines |
| [x] | [ ] | Palaeontology and archaeology |
| [x] | [ ] | Animals and other organisms |
| [x] | [ ] | Clinical data |
| [x] | [ ] | Dual use research of concern |
| [x] | [ ] | Plants |

## Methods

| n/a | Involved in the study | |
|---|---|---|
| [x] | [ ] | ChIP-seq |
| [x] | [ ] | Flow cytometry |
| [x] | [ ] | MRI-based neuroimaging |

# Antibodies

| | |
|---|---|
| Antibodies used | Rabbit polyclonal anti-C4orf14 (1:1000, Invitrogen; Cat#PA5-57420); Rabbit polyclonal anti-ERAL1 (1:2000, Proteintech; Cat#11478-1-AP); Rabbit polyclonal anti-TFB1M (1:1000, Proteintech; Cat#16604-1-AP); Rabbit polyclonal anti-RBFA (1:1000, Novus Biologicals; Cat#NBP1-88471); Rabbit polyclonal anti-NSUN4 (1:1000, Proteintech; Cat#16320-1-AP); Rabbit polyclonal anti-mtIF2 (1:1000, homemade, provided by P.Rehling); Rabbit polyclonal anti-mtIF3 (1:1000, Proteintech; Cat#14219-1-AP); Mouse monoclonal anti-Calnexin (1:500 000, Proteintech; Cat#66903-1-Ig, clone 2A2C6); Mouse monoclonal anti-SDHA (1:20 000, ThermoFisher Scientific, Cat#459200, clone clone 2E3GC12FB2AE2); Mouse monoclonal anti-GAPDH (1:100 000, Santa Cruz, Cat#sc-32233); Rabbit polyclonal anti-COX1 (1:2000, homemade, provided by P.Rehling); Mouse monoclonal anti-COX2 (1:1000, Abcam, Cat#Ab110258; clone 12C4F12); Rabbit polyclonal anti-ATP6 (1:5000, homemade, provided by P.Rehling); Rabbit polyclonal anti-bS1m (1:1000, Proteintech; Cat#16378-1-AP); Rabbit polyclonal anti-uS5m (1:5000, Proteintech; Cat#16428-1-AP); Rabbit polyclonal anti-uS7m (1:1000, Sigma-Aldrich; Cat# HPA 023007); Rabbit polyclonal anti-uS10m (1:1000, Proteintech; Cat#16030-1-AP); Rabbit polyclonal anti-uS14m (1:1000, Proteintech; Cat#16301-1-AP); Rabbit polyclonal anti-uS15m (1:5000, Proteintech; Cat#17106-1-AP); Rabbit polyclonal anti-uS17m (1:1000, ProteinTech; Cat#18881-1-AP); Rabbit polyclonal anti-mS22 (1:5000, ProteinTech; Cat#10984-1-AP); Rabbit polyclonal anti-mS23 (1:10000, ProteinTech; Cat#18345-1-AP); Rabbit polyclonal anti-mS25 (1:5000, ProteinTech; Cat#15277-1-AP); Rabbit polyclonal anti-mS27 (1:10 000, ProteinTech; Cat#17280-1-AP); Rabbit polyclonal anti-mS29 (1:1000, ProteinTech; Cat#10276-1-AP); Rabbit polyclonal anti-mS34 (1:5000, ProteinTech; Cat#15166-1-AP); Rabbit polyclonal anti-mS35 (1:5000, ProteinTech; Cat#16457-1-AP);Rabbit polyclonal anti-mS37 (1:1000, Proteintech, #11728-1-AP); Rabbit polyclonal anti-mS40 (1:5000, ProteinTech; Cat#16139-1-AP); Rabbit polyclonal anti-uL13m (1:5000, Proteintech; Cat#16241-1-AP); Rabbit polyclonal anti-uL23m (1:10 000, homemade; provided by P. Rehling);Rabbit polyclonal anti-POLRMT (1:1000, Proteintech; Cat#17748-1-AP); Rabbit polyclonal anti-LRPPRC (1:2000, Proteintech; Cat#21175-1-AP); Goat IgG anti-rabbit IgG (H+L)-HRPO (1:5000, dianova, #111-035-144); Goat IgG anti-mouse IgG (H+L)-HRPO (1:5000, dianova, #115-035-146); IRDye 800CW Donkey anti-mouse (1:10 000, LI-COR, 926-32212); IRDye 800CW Donkey anit-rabbit (1:10 000, LI-COR, 926-32213) |
| Validation | Commercially supplied antibodies were validated by manufacturers by subjecting lysates of multiple human cell lines (e.g. HEK, HeLa, HepG2) or human tissue (e.g. liver) to SDS-PAGE followed by immunoblotting using the respective antibodies. Additionally, all antibodies for mitoribosomal proteins used in this study follow the expected behavior, meaning detection of ribosomal proteins i.) co-migrating with the mitoribosomal particles in sucrose gradients, and ii.) co-purifying during FLAG-immunoprecipitation of mitoribosome complexes.<br>Homemade antibodies were validated in previous studies ( Rabbit polyclonal anti-uL23m and Rabbit polyclonal anti-COX1: Richter-Dennerlein et al., 2016; Rabbit polyclonal anti-ATP6: Nadler et al., 2022) . Rabbit polyclonal anti-mtIF2 was validated by western blot using cell line overexpressing FLAG-tagged mtIF2 (HEK293-Flp-In T-Rex mtIF2-FLAG) |

# Eukaryotic cell lines

Policy information about cell lines and Sex and Gender in Research

| | |
|---|---|
| Cell line source(s) | HEK293-Flp-In T-Rex (Thermo Fisher Scientific; R78007). HEK293-Flp-In-T-Rex MTG3-/- and HEK293-Flp-In-T-Rex ERAL1-/- cell lines were generated using CRISPR/Cas9 technology. HEK293Flp-In-T-Rex MTG3-FLAG, HEK293-Flp-In-T-Rex MTG3-/-+ MTG3-FLAG, HEK293-Flp-In-T-Rex MTG3-/- + dN-MTG3-FLAG, HEK293-Flp-In-T-Rex MTG3-/-+ MTG3-G499P-FLAG, HEK293-Flp-In-T-Rex ERAL1-/- + ERAL1-FLAG and HEK293-Flp-In-T-Rex mtIF3-FLAG cell lines were generated by co-transfecting the HEK293-Flp-In T-Rex WT cell line with pcDNA5/FRT/TO bearing the respective FLAG tagged nucleotide sequence and pOG44 Flp Recombinase Expression Vector |
| Authentication | CRISPR/Cas9 mediated knockout of MTG3 and ERAL1 were confirmed by western blotting and by sequencing. Stable insertion of the FLAG-Tagged proteins were confirmed by western blotting using mouse monoclonal anti-FLAG (Sigma Aldrich) antibody |
| Mycoplasma contamination | Cell lines used in this study were systematically tested negative for the presence of Mycoplasma by GATC Biotech. |
| Commonly misidentified lines<br>(See ICLAC register) | No commonly misidentified cell lines were used. |

# Plants

| | |
|---|---|
| Seed stocks | *Report on the source of all seed stocks or other plant material used. If applicable, state the seed stock centre and catalogue number. If plant specimens were collected from the field, describe the collection location, date and sampling procedures.* |
| Novel plant genotypes | *Describe the methods by which all novel plant genotypes were produced. This includes those generated by transgenic approaches, gene editing, chemical/radiation-based mutagenesis and hybridization. For transgenic lines, describe the transformation method, the number of independent lines analyzed and the generation upon which experiments were performed. For gene-edited lines, describe the editor used, the endogenous sequence targeted for editing, the targeting guide RNA sequence (if applicable) and how the editor was applied.* |
| Authentication | *Describe any authentication procedures for each seed stock used or novel genotype generated. Describe any experiments used to assess the effect of a mutation and, where applicable, how potential secondary effects (e.g. second site T-DNA insertions, mosiacism, off-target gene editing) were examined.* |
